# Defining a core breath profile for healthy, non-human primates

**DOI:** 10.1038/s41598-024-64910-y

**Published:** 2024-07-23

**Authors:** Carly A. Bobak, Keisean A. J. M. Stevenson, Ning Sun, Mohammad S. Khan, Jannatul Azmir, Marco Beccaria, Jaime A. Tomko, Daniel Fillmore, Charles A. Scanga, Philana L. Lin, JoAnne L. Flynn, Jane E. Hill

**Affiliations:** 1 Department of Chemical and Biological Engineering, University of British Columbia, Vancouver, V6T 1Z3 Canada; 2 Thayer School of Engineering, Dartmouth College, 14 Engineering Drive, Hanover, NH 03755 USA; 3 School of Biomedical Engineering, University of British Columbia, Vancouver, V6T 1Z3 Canada; 4 Department of Microbiology and Molecular Genetics, University of Pittsburgh School of Medicine, Pittsburgh, PA USA; 5 Department of Pediatrics, Division of Infectious Disease, Children’s Hospital of UPMC, University of Pittsburgh School of Medicine, Pittsburgh, PA USA; 6 Present Address: Department of Biomedical Data Science, Dartmouth Geisel School of Medicine, Lebanon, NH USA; 7 Present Address: Department of Chemistry, University of Winnipeg, Winnipeg, MB Canada; 8 Present Address: Cargill Inc., Wayzata, MN USA; 9 Present Address: Department of Chemical, Pharmaceutical, and Agricultural Sciences, University of Ferrara, 44121 Ferrara, Italy

**Keywords:** Metabolomics, Health care, Mass spectrometry

## Abstract

Non-human primates remain the most useful and reliable pre-clinical model for many human diseases. Primate breath profiles have previously distinguished healthy animals from diseased, including non-human primates. Breath collection is relatively non-invasive, so this motivated us to define a healthy baseline breath profile that could be used in studies evaluating disease, therapies, and vaccines in non-human primates. A pilot study, which enrolled 30 healthy macaques, was conducted. Macaque breath molecules were sampled into a Tedlar bag, concentrated onto a thermal desorption tube, then desorbed and analyzed by comprehensive two-dimensional gas chromatography-time of flight mass spectrometry. These breath samples contained 2,017 features, of which 113 molecules were present in all breath samples. The core breathprint was dominated by aliphatic hydrocarbons, aromatic compounds, and carbonyl compounds. The data were internally validated with additional breath samples from a subset of 19 of these non-human primates. A critical core consisting of 23 highly abundant and invariant molecules was identified as a pragmatic breathprint set, useful for future validation studies in healthy primates.

## Introduction

Non-human primates (NHPs) are frequently used in biomedical research as they share anatomy and physiology similar to humans^1–3^, including their response to diseases and therapeutics^4–7^. For example, NHPs have been shown to exhibit a similar spectrum of clinical and pathological characteristics observed in human tuberculosis (TB)^8^. NHPs have also been used extensively in SARS-CoV2 research over the past 3 years, including for therapeutics and vaccines^9,10^. These animals are sentient and phylogenetically advanced, so their research use is restricted and highly regulated^11–13^. When their use in research is deemed necessary, minimally invasive procedures are highly desirable. Current methods use blood, bronchoalveolar lavage fluid, or positron emission tomography–computed tomography (PET–CT) scans to monitor macaque whole or local health status, especially in lung disease models, which are either invasive or expensive. However, breath collection is less invasive, only requires intubation under light sedation with free breathing, and takes 3–5 min^14^. Thus, exhaled breath analysis is a new approach that could not only assist scientific progress but also reduce research animal use and trauma.

Apart from exogenous compounds, exhaled breath includes molecules created from normal metabolism, microbiomes, and disease-specific processes^15,16^. There are published breathprints for infectious diseases, cancers, and metabolic disorders in humans^17–20^, so it stands to reason that the breath of NHPs can also be used to monitor their health. Take lung infectious disease as an example, as the lung is the reservoir and origin of the breath and exchanging health information via blood. Only two studies involving NHPs have identified a potential breathprint differentiating between macaques infected with *Mycobacteria tuberculosis* (*Mtb*) and those uninfected with *Mtb*, utilizing two-dimensional gas chromatography with time-of-flight mass spectrometry (GC × GC-ToFMS). One of the studies showed that 37 volatile compounds produced by in vitro-grown *Mtb* were detectable in the breath of macaques^21^. The other study putatively identified 19 compounds that expressed higher in either preinfection or postinfection samples, and three of them (Dodecane, Hexylcyclohexane, and Tridecane) overlapped with human *Mtb* breath biomarker^14^, indicating that NHP breath volatile organic compounds (VOCs) can not only monitor their health but also can be a bridge to human research. On the other hand, baseline studies confirming the presence of the molecules in healthy NHPs and measuring the range of the molecules in healthy status are critical for monitoring the concentration of these compounds in disease conditions. To the best of our knowledge, no such studies have been published. As a first step towards developing a breath baseline in NHP research, we share here our approach to defining the pan volatilome and a group of consistent, prevalent breath molecules in captive, healthy macaques.

## Results and discussions

### Defining the breath volatilome of non-human *primates*

**Figure 1:**
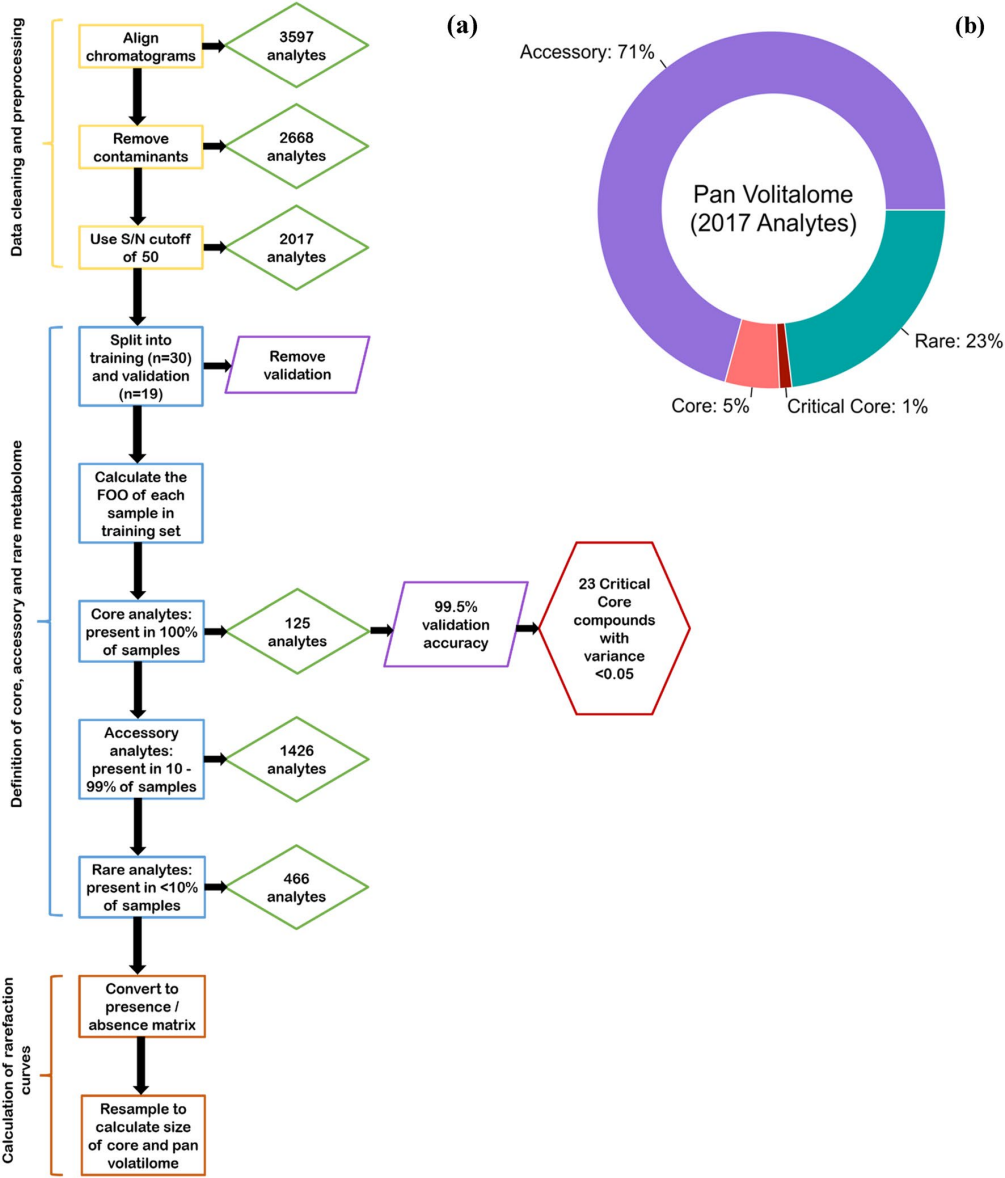
(**a**) The data analysis process and reduction in analytes as they are removed or classified according to core, critical core, accessory, or rare status. (**b**) The breakdown of the proportion of molecules in the pan volatilome that belong to each subclass.

**Figure 2:**
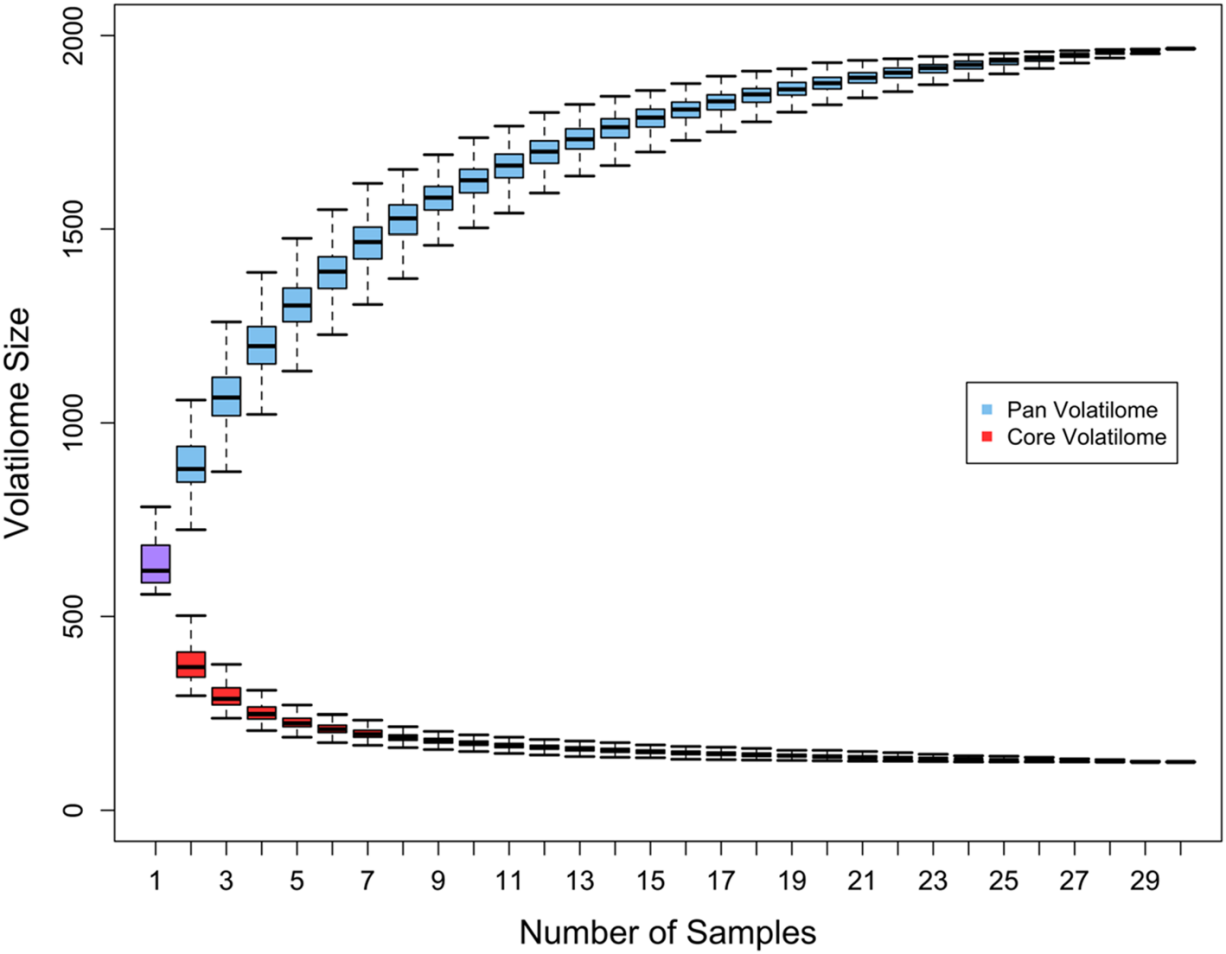
Accumulated and rarefaction curves generated from random sampling without replacement 500 times from the training dataset and examining the size of the resulting pan and core volatilomes as a function of sample size.

In this pilot study, we collected and analyzed the breath of 30 healthy NHPs and found 2,017 volatile features in their collective breath samples (Fig. 1). Of these, 125 were consistently found in the breath from every animal in this study, which we define as the core breathprint (supplemental Tables S2, S3, and S4). We define their accessory breath volatilome (i.e., features present in more than 10% of samples but less than 100%) as having 1,426 features. We define sparse features present in less than 10% of the samples as the rare breathprint, consisting of 466 features. We conducted an accumulation and rarefaction curve analysis (Fig. 2) to assess whether 30 animals were enough to capture the full diversity of molecules in this macaque species under our experimental conditions, an approach commonly used in genetic studies^22^. Each curve’s asymptote indicates that the pan and core breathprint sizes converged (Fig. 2). Thus, the 30 animals from which breath was sampled capture the full variation of the pan and core breathprint size, and breath samples from additional animals would likely not significantly change the results^22,23^. The core molecules comprise aliphatic hydrocarbons (primarily alkanes, alkenes, and terpenes), accounting for 40% of the core profile. Aromatic compounds (benzene derivatives, naphthalene, and furan) represented 28% of the core profile. Although classified as aromatic, some compounds in this class may also fall into the third largest category, carbonyl compounds. This group comprised 16% of the core profile and included non-aromatic aldehydes, ketones, and esters. A complete list of core molecules, retention times, linear retention index (LRI), and putative annotations are shown in Tables S2, S3, and S4. Broadly speaking, healthy human breath contains over 1,000 separate molecules, including hydrocarbons, benzene derivatives, aldehydes, ketones, esters, and alcohols^24–28^. Hydrocarbons represent the most considerable fraction of volatiles in human breath, which is consistent with what we found in macaques. Healthy human breath also contains trace amounts of amino, nitrile, acids, and sulfide groups^26,27^, which we also observed in healthy macaques’ accessory and rare breathprints. However, some high prevalence features in human breath were not reported as core molecules in macaque breath. The untargeted GC method aims to detect as many volatile organic compounds as possible, while an unoptimized GC method may cause peak split, overlap, or even undetection. For example, 2-pentanone was included in accessory volatilome (only absent in one sample). Acetone was detected in the early stage of the chromatograph with broad and splitted peaks due to the effect of moisture in the breath sample, so the frequency of observation was low. Isoprene was not detected in our method because of its low molecular weight and early elution time, so the software could not identify the peak. Therefore, optimization of the GC method is vital in the future to increase the reliability of peak identification, especially for targeted and quantitative analysis.

### Validation of the core, accessory, and rare volatilomes

As an initial step in validating these core, accessory, and rare volatilome results, we examined the frequency with which we observed these features in an additional 19 samples from the identical 30 macaques on different sampling days used as a validation set. Regarding the core breathprint, and in accordance with our S/N cut-offs, we initially found that one core molecule was not observed in four validation samples, four core molecules were not observed in two validation samples, and 22 core molecules were not observed in one validation sample. This meant that 27 core molecules (based on the training set) were missing, with varying frequency, across seven samples. However, a careful visual comparison of the mass spectra of these “missing” peaks (using ^1^D and ^2^D retention times that fall within 6.0 s and 0.3 s of known core molecules) revealed that many were indeed present but not assigned a putative identification by ChromaToF. Following these checks, we found that:

- Only 12 core molecules in the training set were not found in the validation set
- Each missing molecule was undetected in only one sample
- One breath sample, denoted S26, accounted for seven of the 12 missing molecules

Ultimately, 113 of the 125 core molecules defined in the training set were observed in 100% of the validation samples. Table S6 shows the 12 missing core molecules and their distribution across samples. Of the five validation samples for which only one core molecule was missing, four samples (S14, S24, S40, and S42) came from male Chinese cynomolgus macaques, while one (S4) was from a female Chinese cynomolgus macaques (Table S1). Given the small sample sizes (only 4 females to 26 males), narrow age range (6–8 years), and the detection of these molecules in the other sample from these animals, we are unable to attribute these missing observations to any of these factors meaningfully.

Additionally, the pattern in Table S6 suggests something unusual about sample S26. The frequency of missing core molecules (7/12) relative to every other sample with missing core molecules (1/12) implies that the features are “Missing Not at Random” for S26. Due to the more anomalous nature of S26, we checked the retention times and peak areas of its core molecules that were not missing and did not find anything that would allow us to classify it as an outlier. We also note that the animal from which sample S26 was taken was very similar to 16 other animals in the study in terms of species, origin, age, and sex (i.e., *Macacca fascicularis*, Chinese, six years old, and male). Therefore, these factors also did not provide any information that would allow us to confidently call this sample an outlier. As such, although removing this sample would yield a higher Validation Accuracy, we have kept it in our calculation. Using Equation (1), we determined a Core Validation Accuracy of 99.5% (Fig. 1).

It is important to note that this number does not reflect the portion of the core molecules from the training set that were found in 100% of the validation set but instead reflects the number of times core molecules were observed in validation samples relative to the expected number of observations. This Core Validation Accuracy reveals that core molecules defined by the training set were observed in the validation set in 99.5% of expected instances.

This represents a very high validation accuracy despite the inclusion of two female Indian rhesus macaques (animals A5 and A6) in the core-defining training set. This suggests that the core breath volatilome, for this cohort at least, may be common across members of the same genus, regardless of origin, sex and species. Confirming this will require thorough evaluation with a larger cohort, which includes a more heterogeneous distribution of macaque species, origin, age, and sex, as well as multiple samples from individuals in each of these categories. This would allow the use of more varied training and validation sets.

An evaluation of the accessory breathprint showed that of the 1,426 accessory features, 1,199 were found in between 10 and 100% of the samples in the validation data. Thirty-one accessory features were observed in 100% of the validation samples; 124 accessory features were found in only one validation sample (5.26%), and 72 were not found in the validation data. Of these accessory features, 142 were significantly different in frequency of observation across the training and validation data using a Fisher’s exact test where $\alpha =0.05$. For the rare features, 382 of the 466 features did not significantly differ between the training and validation data at a significance threshold of $\alpha =0.05$. Given the large number of features found in these sets, as well as the relatively high level of confidence in the spectral matching of the core molecules, no mass spectral checks were done to confirm the validity of the absence of “missing” accessory and rare peaks.

### Identification of the critical core breathprint

Routine animal health monitoring that requires measuring 125 molecules is impractical for most settings. Therefore, we identified a subset of the core molecules that have the smallest variance for easier translation to more standard GC–MS systems or equivalent (Fig. S1). These 23 molecules, which have a normalized area variance < 0.05 across the entire cohort of macaques, are termed the ‘critical core’ breathprint for healthy macaques. Notably, all but one of these molecules has a higher-than-average normalized peak area compared to those molecules in the accessory and rare volatile, indicating that the 23 critical core components are abundant as well as consistently invariant. These molecules are reliably present across each of the 49 macaque breath samples analyzed here.

**Figure 3:**
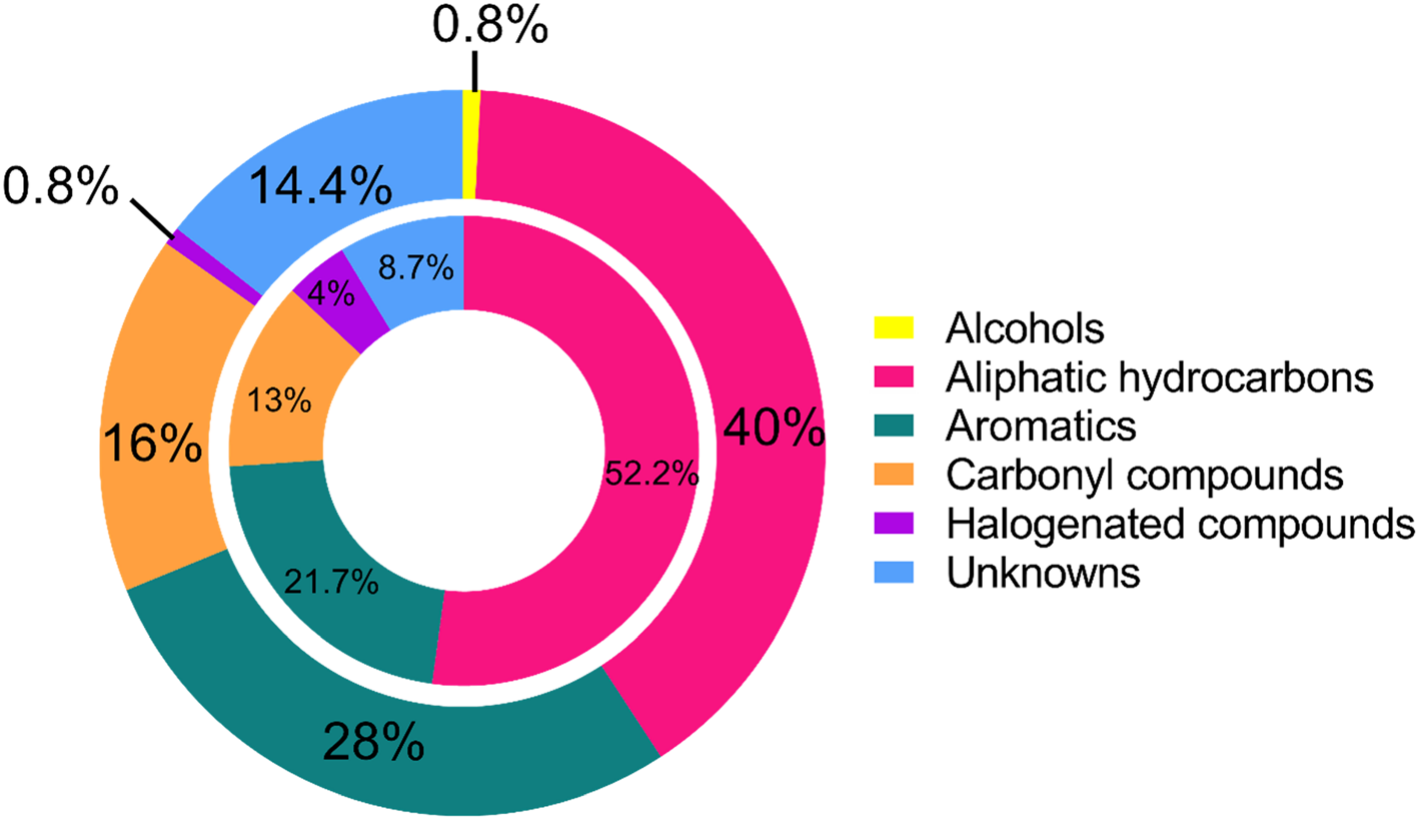
The outer chart shows the compound class distribution of the 125 core molecules while the inner chart represents the compound class distribution of the 23 critical core molecules. Note that compounds that showed aromaticity along with other functionalities (e.g., acetophenone, an aromatic ketone) were grouped as aromatics. Aldehydes, ketones, and esters were grouped collectively as carbonyl compounds.

We applied the proposed Metabolomic Standards Initiative criteria to the 23 critical core peaks^29^. Therefore, we can provide putative names for two molecules (Level 2), putative formulae (without name) for nine molecules and a putative class (without name or formula) for ten molecules (Level 3). No putative identification could be confidently given for two of the peaks, though their presence and reproducibility are confirmed (Level 4). Table 1 lists these core molecules along with their mean retention times on both columns. Aliphatic hydrocarbons constitute the majority of this critical core (52.2%, 11 alkanes, and one terpene), while aromatic compounds (21.7%, four benzene derivatives, and one furan) represented the second largest compound class. Figure 3 illustrates the distribution of the compound classes that comprise the critical core alongside the distribution of the entire core subset of 125 compounds. A comparison of the molecular formulae of compounds identified in this current study with that in Bishop et al*.*^30^, which reported the exhaled volatiles associated with cardio-metabolic health in baboons, revealed 15 matching formulae. Further, of the seven compounds in this study to which putative names could be assigned, two matched putative names were given in the Bishop et al*.* study. These 17 compounds (15 matched by formulae and two by putative name) are listed in supplemental Table S5.

**Table 1:** List of 23 critical core compounds. Names, formulae, and class assignments are putative.

Annotation	^1^t̄_R_ (s)	^2^t̄_R_ (s)	Putative class	LRI	Top 4 *m/z*	Average area ratio (breath/room air)
C_7_H_16_	612	0.55	Alkane	673	43, 70, 56, 100	1.0
C_7_H_16_	674	0.56	Alkane	700	43, 57, 71, 100	1.0
Toluene	936	0.75	Aromatic	790	91, 39, 65, 51	0.9
C_6_H_12_O	1083	0.75	Aldehyde/ketone	838	44, 56, 72, 82	3.0
C_7_H_14_O	1399	0.75	Aldehyde/ketone	941	44, 55, 70, 81	2.4
C_10_H_16_	1417	0.60	Terpene	947	93, 39, 77, 51	6.8
C_9_H_14_O	1600	0.71	Furan	1009	81, 53, 39, 138	3.6
Unknown 32	1604	0.56	Alkane	1010	43, 71, 57, 85	6.5
Octanal	1704	0.74	Aldehyde	1044	41, 57, 84, 69	2.2
Unknown 40	1826	0.67	Chloroalkane	1086	91, 41, 55, 69	3.3
C_11_H_20_	1965	0.63	Aromatic	1137	81, 67, 41, 95	1.8
Unknown 44	2124	0.60	Aliphatic hydrocarbon	1196	41, 55, 69, 82	1.7
C_12_H_26_	2135	0.59	Alkane	1200	43, 57, 71, 85	3.8
C_10_H_8_	2258	1.12	Aromatic	1248	128, 51, 102, 63	2.0
Unknown 48	2360	0.57	Alkane	1289	57, 43, 71, 85	0.6
Unknown 50	2389	0.58	Alkane	1300	43, 57, 71, 85	0.8
Unknown 13	2400	0.53	Unknown	1305	73, 45, 59, 147	3.8
Unknown 52	2531	0.58	Alkane	1359	43, 57, 71, 85	1.0
Unknown 53	2562	0.58	Alkane	1372	57, 41, 71, 85	1.8
Unknown 54	2576	0.58	Alkane	1378	57, 43, 71, 85	1.8
Unknown 55	2604	0.60	Alkane	1390	41, 55, 70, 83	0.6
Unknown 56	2628	0.59	Alkane	1400	43, 57, 71, 85	1.8
Unknown 14	2652	0.58	Unknown	1411	57, 43, 71, 85	2.8

For unknowns 13 and 14, no class could be confidently proposed.

*LRI:* Linear retention index.

To investigate the difference between a critical core molecule in breath and that in the environment (room air), we independently identified and aligned 18 room air samples. All critical core molecules were present in all room air samples, but the average peak area ratios between the two samples (breath sample/room air sample) were different (Table 1). A total of 16 compounds were enriched in the breath samples, ranging from 1.7 to 6.8 times higher than in room air samples, suggesting that they were potential real baseline compounds. Three out of 23 compounds (unknown 48, 50, 55) had a ratio of less than one, ranging from 0.6 to 0.8, suggesting that they may be enriched in the room air samples. Another four compounds have a ratio of one, indicating that similar amounts were present in both breath and room air samples. It makes sense that exogenous compounds are exchanged consistently when the monkey is breathing in a fixed room. However, there were some limitations in this comparison. Due to the separate alignment and data processing, we were only able to use the average peak area to compare the difference between breath and background, which had higher variability compared with normalized peak areas, let alone to the possibility of difference in the quantitative mass selected by the software for peak area calculation (such as the three compounds enriched in the room air samples). Aligning all samples together may solve this issue in the future and can compare all compounds automatically instead of focusing on critical core molecules manually. In addition, breath VOCs are highly variable, and 5% normalized peak area variance could restrict criteria in selecting the critical core molecules and further reduced the numbers of selected critical core molecules after comparing the average peak areas between breath and room air. However, we respectfully suggest that it is not clear how best to use room air samples. In this BSL3 facility, for example, the air exchange is substantial while we recognize that there is an exchange in air and monkey lungs, which gives us high variance in the peak area intensities. Therefore, even some compounds were enriched in room air samples, we still kept them as critical core molecules in this pilot study.

In addition, the linear retention index (LRI) is commonly used for reporting chemical compounds. Since no references were analyzed along with our samples, we can not calculate the LRI directly. However, breath compounds were predominant in hydrocarbons, indicating that a series of n-alkanes could be present in the samples. Therefore, we checked all compounds via the putative names provided by the software, mass spectral information for alkanes (such as a series of 14 differences between two fragments), and retention times compared with alkane reference standards analyzed in other studies but with the same GC conditions on different days. As such, we can generate a calibration curve (C6–C18 in this study) to calculate the LRI indirectly. The LRI for critical core molecules was shown in Table 1, along with the top 4 mass spectral information (sorted in descending order of abundance). Based on the LRI and mass spectral data, we have more confidence in identifying some of the compounds. For example, the alkane with formula C_7_H_16_ and LRI 700 could be heptane; the unknown 50 with LRI 1300 and four m/z fragments indicate a n-tridecane; the unknown 56 with LRI 1400 and four m/z fragments indicate a n-tetradecane. Due to the column type (Rxi-624Sil MS, mid-polar), the LRI of the compounds was not able to match the NIST library. However, it was expected between the LRI obtained from non-polar and polar GC columns. Hence, the annotation for core molecules was still only defined by the criteria described in S2 of the supplementary material.

The work in this pilot study describes a methodology for analyzing exhaled volatiles in NHPs and represents the initial effort in establishing distinctive breath signatures that characterize healthy NHPs. The core breathprint mainly comprises aliphatic hydrocarbons, aromatic compounds, and carbonyl compounds. The critical core breathprint consists of 23 highly abundant and invariant molecules, which can serve as a basis for further validation studies. Research based on these data will need to incorporate external validation of these analytes. This involves using internal or external chemical authentication standards to validate the identity of the breathprint, especially for the core or critical core molecules. This also involves adding quality control samples to monitor the quality of breath collection (such as the impact of sedation), the shipping and storage, and the stability of the breath samples. In addition, for the purposes of study improvement, it is also necessary to increase the heterogeneity of the animal and the sample size to have better statistical power, such as age- and gender-matched rhesus macaques in this study. Collecting breath samples longitudinally is also an option to prove intra-primate reproducibility. As a baseline compound, their presence or amount should be kept steady or reasonable. Therefore, future studies aimed at expanding the repertoire of NHP VOCs, assessing the analytical and biological sensitivity, and quantifying the range of these compounds under various conditions (e.g., diet, disease status, etc.) will provide valuable insights.

## Methods

### Animals and design

In this pilot study, a total of 49 breath samples were collected from 30 healthy macaques (28 cynomolgus macaques—*Macaca fascicularis* and two rhesus macaques—*Macaca mulatta*) housed in an AAALAC-accredited facility at the University of Pittsburgh (see Supplemental Table S1 for information on species, place of origin, age, and sex of the animals). Animals were obtained from Valley Biosystems (West Sacramento, CA), Buckshire Corporation (Exton, PA), California Primate Center (Davis, CA), and BIOQUAL (Rockville, MD). Macaques were pair-housed in rooms with autonomously controlled temperature, humidity, and lighting. They were fed twice daily with biscuits formulated for NHP, supplemented at least four days/week with fresh fruits, vegetables, or other foraging mix, with ad libitum access to water. An enhanced enrichment plan, overseen by an NHP enrichment specialist, was in place as previously described^31^. Prior to admission to the study, each animal underwent routine testing, including a physical exam, body weight, complete blood count, and blood chemistry panel to verify that they were free of specific infections or diseases that might confound outcomes of the intended experimental infection or host response (e.g., SIV, helminth or intestinal pathogen). Once on-study, animals were checked at least twice daily to assess appetite, activity level, hydration status, etc. Physical exams, including weight assessment, were done at least monthly. All animal protocols and procedures were approved by the University of Pittsburgh’s Institutional Animal Care and Use Committee (IACUC) which adheres to guidelines established in the Animal Welfare Act and the Guide for the Care and Use of Laboratory Animals, as well as the Weatherall Report (8th Edition). The University is fully accredited by AAALAC (accreditation number 000496), and its OLAW animal welfare assurance number is D16-00118 (A3187-01). The animals included in this study were covered under IACUC protocol numbers 13122856 (approved 2013), 15035407 (approved 2015), 15066174 (approved 2015), and 15126588 (approved 2015). All methods were performed in accordance with relevant guidelines and regulations. All animal experiments complied with the ARRIVE guidelines.

### Breath collection

The methods used for breath collection and breath sample concentration were described in detail previously^14^. In short, animals were sedated via an intramuscular injection of ketamine (10–20 mg/kg, Covetrus, Portland, ME) and briefly intubated with a low-pressure cuffed endotracheal tube. While freely breathing, their breath was collected into a 5-L Tedlar bag (SKC, Eighty Four, PA) over the course of 3–5 min, resulting in the collection of up to 4 L of breath. Breath was moved via an air sampling pump (AirChek TOUCH Pump, SKC, Eighty Four, PA, USA) at a rate of 150 mL/min and concentrated for 10 min onto two three-bed thermal desorption (TD) tubes containing Carbopack Y, Carbopack X, and Carboxen 1000 (Supelco, Bellefonte, PA). 1.5L of room air samples were also collected on the same day in the same room for breath collection. TD tubes containing breath molecules were hermetically sealed and stored at 4 °C until further analysis, which occurred within a month from collection, as previously reported^32^.

### Sample analysis and data processing

The instrumental conditions for sample loading, injection, and analysis were summarized previously^14^. Briefly, one of the TD tubes from each subject was introduced by a rail autosampler (MPS, Gerstel, Linthicum Heights, MD), and breath molecules were desorbed using a Gerstel TDU system in splitless injection mode. Separation and analysis were performed using a GC × GC-ToF MS instrument (LECO, St. Joseph, MI). The blank samples (clean TD tube) were analyzed before each batch to investigate system contaminations. Detailed instrumental parameters are described in the supplemental information section S1. Chromatographic data was processed and aligned using the Statistical Compare feature of ChromaTOF (LECO, St. Joseph, MI). A signal-to-noise (S/N) cutoff was set at 100:1 in at least one chromatogram, and 20:1 in all others, and resulting peaks were putatively identified by comparison with the National Institute of Standards and Technology (NIST) 2011 library. For chromatographic peak alignment, maximum deviations of first- (^1^D) and second- (^2^D) dimension retention time were 2.0 s and 0.1 s, respectively, while the spectral match threshold was set to 600.

To further confirm that the features were well matched across aligned samples, the mass spectra of each molecule that was present in all 30 test set samples (core molecules) were visually inspected. During this check, the *m/z* and relative intensities of the four most abundant peaks of a given core compound were compared across test samples. The overall distribution of *m/*z and relative intensities of lower abundance peaks were also compared. While the four most abundant peaks, along with most other peaks at expected *m/z* channels, were present for each core molecule in all samples, the relative intensities of these peaks were not always consistent. This was most commonly seen with *m/z* 57 and 71 for suspected alkanes and was also observed, though less frequently, with mass channels 91 and 119 for suspected aromatic compounds. Despite these differences, the core molecule spectra were similar enough in all other respects, and the chromatographic peaks fell within narrow retention time windows across samples. As such, all core compounds in question were considered to be well-aligned.

Linear retention index (LRI) was calculated for all compounds based on the internal n-alkanes identified by visual inspection (putative names from the ChromaTOF software, m/z channels in the experimental spectra) and comparison with reference standards analyzed in another project on different days using the same GC method. A calibration curve was generated for extrapolation of compounds eluted before the first alkane or after the last alkane.

### Statistical analysis

All statistical analyses were performed using R version 3.6.1 (R Foundation for Statistical Computing, Vienna, Austria). Before splitting the dataset into training (n = 30) and validation (n = 19) sets, contaminants were removed from the dataset^33^, and S/N thresholding was set to 50:1. The relative abundance of the remaining features across chromatograms was first normalized using Probabilistic Quotient Normalization (PQN) and log_10_ transformed^34^. All remaining features after S/N thresholding and artifact removal are classified as part of the pan breathprint which consists of three subclasses: core, accessory, and rare. These subclasses are defined according to the following criteria and calculated by frequency of observation (FOO) count: core breath molecules are those found in all samples (n = 30), rare breath features are identified as those detected in ≤ 10% of samples (n ≤ 3), and accessory breath features are identified as those present in > 10% and < 100% of samples (3 < n < 30). The core molecules were identified at Levels 2, 3, or 4, according to the Metabolomics Standards Initiative criteria^29^, based on comparing their experimental spectra to the NIST 2011 library. The mass spectral matching criteria used to assign compounds to a given putative identification level are described in the supplemental information (Section S2). We also defined a subset of the core, which we termed the critical core. It consists of molecules with normalized peak areas that were higher than average and whose normalized peak variances were < 0.05 across all 49 samples analyzed.

Accumulation and rarefaction curves were created using a methodology previously described by Humbert and colleagues for analyzing genomic data^35^ and used by Bean and colleagues for analyzing volatile metabolomic data^23^. A binary scale was generated to describe the presence or absence of volatile features in each breath sample (0 for absent; 1 for present). A total of 500 iterations were performed to formulate the accumulation and rarefaction curves using a subset of data selected at random and without replacement.

To validate our findings in the core, accessory, and rare volatilome subclasses, we compared the proportion of observed peaks in the training data to the proportion of observed peaks in the validation data. Beyond a manual comparison that the analytes were observed and classified according to the same subclass in both the training and the validation datasets, Fisher’s exact test^36^ was used to quantitively compare the proportion of samples in which the analytes were observed across the training and validation datasets. A complete summary of the data processing and statistical analysis steps is shown in Fig. 1a. Further, for the core analytes, we calculated a Validation Accuracy according to the following equation:

$$Validation Accuracy = 100 \times \frac{{\mathop \sum \nolimits_{i = 1}^{{a_{t} }} (n_{v} - n_{miss} )}}{{n_{v} \times a_{t} }}$$

where *n*_*v*_ = number of validation samples, *n*_*miss*_ = number of samples in which core feature, *i,* was not observed, and *a*_*t*_ = number of unique core molecules found in the training samples.

### Supplementary Information

Supplementary Information.

## Data Availability

The dataset used and analyzed during the current study is available from the corresponding author upon reasonable request.
