# Supplementary material for: Defining a core breath profile for healthy, non-human primates
Source: Sci Rep. 2024 Jul 23;14:17031. doi: 10.1038/s41598-024-64910-y (PMC11266492; doi:10.1038/s41598-024-64910-y)

**S1: Detailed instrumental conditions**

The two-dimensional column set consisted of a Rxi-624Sil MS column (60 m × 250 μm × 1.4 μm (length × internal diameter × film thickness); Restek, Bellefonte, PA) in the first dimension and a Stabilwax column (1.5 m × 250 μm × 0.5 µm; Restek) in the second dimension. The samples were introduced via a Gerstel TDU system in splitless injection mode with solvent venting performed for 10 min at 50°C (flow rate of 75ml/min) and thermal desorption performed for 5 min at 330°C (flow rate of 75 mL/min). The samples were cryo-focused for 5 min at -120°C before injection into a 275°C injector port for 3 min. Primary oven temperature was programmed as follows: 35°C (0.20 min hold), then ramped at 3.5 °C/min to 235 °C (5 min hold). A +5°C secondary oven offset (relative to the primary oven) was used throughout the run, while a +15°C modulator offset was chosen. The modulation period was 2 s with a 0.5 s hot pulse. A helium carrier gas flow rate of 3 mL/min (constant flow mode) was used. This method resulted in a run time of 62.2 min per sample. Mass spectra were acquired over the range of 30 to 500 m/z at a rate of 200 spectra/s. The ion source temperature was set to 200°C, and electron impact ionization was done at -70 eV. Data acquisition and analysis were performed with ChromaTOF software, version 4.50 (LECO).

**S2: Putative annotation and class assignment of core compounds**

Putative annotation of the core molecules was performed using the NIST 11 EI Mass Spectral Library (NIST) with a minimum similarity match of 700 for assigning names (in the software). This similarity match, however, was not the only factor considered in the annotation of core molecules presented in this paper. Molecules were split into four categories shown in the consort diagram below: A) those assigned a putative name, B) those assigned a putative molecular formula (but no name), C) those assigned a putative class only, and lastly, D) those that remained unknown.


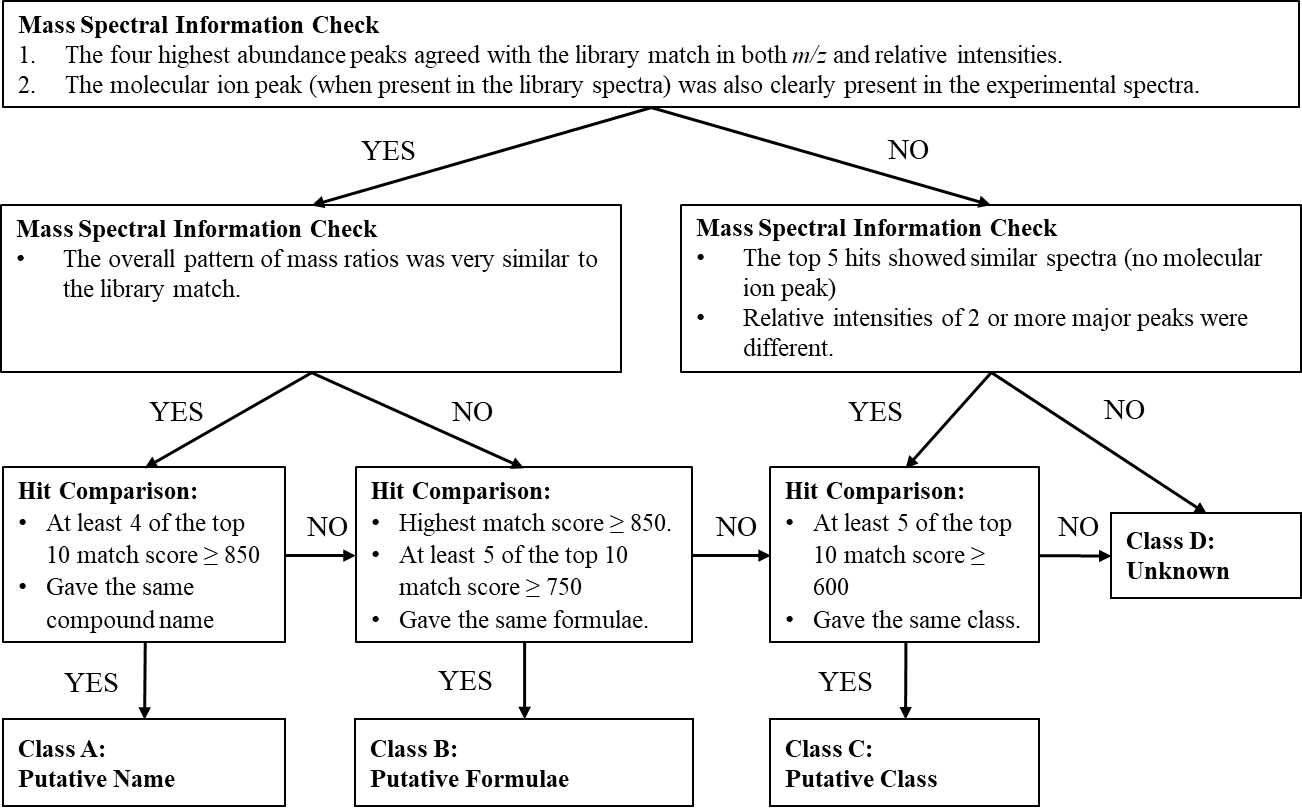


**Table S1**: Information on the animals used in this study. Sample IDs and Animal IDs shown here were assigned using a simple numbering system to aid the generation and readability of this table. They do not reflect the order in which samples were run nor the identification numbering system used for the animals.

| **Animal ID** | **Sample IDs** | **Species** | **Origin** | **Age (y)** | **Sex** | **Date of**  **Breath**  **Collection**  **(MM/DD/YYYY)** | **Date of Sample Analysis**  **(MM/DD/YYYY)** |
| --- | --- | --- | --- | --- | --- | --- | --- |
| A1 | S2 | *Macaca fascicularis* | Mauritius | 7 | F | 10/30/2015 | 11/18/2015 |
| A2 | S3, S4 | *Macaca fascicularis* | Mauritius | 8 | F | 10/30/2015; 11/12/2015 | 11/18/2015 |
| A3 | S5, S6 | *Macaca fascicularis* | Mauritius | 6 | M | 10/30/2015; 11/12/2015 | 11/18/2015 |
| A4 | S7, S8 | *Macaca fascicularis* | Mauritius | 6 | M | 11/02/2015; 11/12/2015 | 11/18/2015 |
| A5 | S9 | *Macaca mulatta* | Indian | 6 | F | 10/12/2015 | 11/18/2015 |
| A6 | S10 | *Macaca mulatta* | Indian | 7 | F | 10/12/2015 | 11/18/2015 |
| A7 | S11 | *Macaca fascicularis* | Chinese | 8 | M | 11/10/2015 | 11/18/2015 |
| A8 | S12 | *Macaca fascicularis* | Chinese | 6 | M | 11/10/2015 | 11/18/2015 |
| A9 | S13, S14 | *Macaca fascicularis* | Chinese | 7 | M | 11/23/2015; 12/10/2015 | 12/19/2015 |
| A10 | S15, S16 | *Macaca fascicularis* | Chinese | 7 | M | 11/23/2015; 12/10/2015 | 12/19/2015 |
| A11 | S17, S18 | *Macaca fascicularis* | Chinese | 6 | M | 11/19/2015;12/08/2015 | 12/19/2015 |
| A12 | S19, S20 | *Macaca fascicularis* | Chinese | 6 | M | 11/19/2015; 12/02/2015 | 12/19/2015 |
| A13 | S21, S22 | *Macaca fascicularis* | Chinese | 6 | M | 11/19/2015; 12/02/2015 | 12/19/2015 |
| A14 | S23, S24 | *Macaca fascicularis* | Chinese | 6 | M | 11/19/2015; 12/02/2015 | 12/19/2015 |
| A15 | S25, S26 | *Macaca fascicularis* | Chinese | 6 | M | 11/19/2015; 12/02/2015 | 12/19/2015 |
| A16 | S27, S28 | *Macaca fascicularis* | Chinese | 6 | M | 11/19/2015; 12/02/2015 | 12/19/2015 |
| A17 | S29 | *Macaca fascicularis* | Chinese | 7 | M | 6/26/2015 | 11/18/2015 |
| A18 | S30 | *Macaca fascicularis* | Chinese | 7 | M | 6/26/2015 | 11/18/2015 |
| A19 | S31 | *Macaca fascicularis* | Chinese | 8 | M | 6/26/2015 | 11/18/2015 |
| A20 | S32 | *Macaca fascicularis* | Chinese | 6 | M | 2/19/2016 | 6/13/2016 |
| A21 | S33 | *Macaca fascicularis* | Chinese | 6 | M | 2/22/2016 | 6/13/2016 |
| A22 | S34 | *Macaca fascicularis* | Chinese | 6 | M | 2/19/2016 | 6/13/2016 |
| A23 | S35, S36 | *Macaca fascicularis* | Chinese | 7 | M | 5/17/2016; 5/26/2016 | 6/13/2016 |
| A24 | S37, S38 | *Macaca fascicularis* | Chinese | 6 | M | 4/5/2016; 4/21/2016 | 6/13/2016 |
| A25 | S39, S40 | *Macaca fascicularis* | Chinese | 6 | M | 4/5/2016; 4/21/2016 | 6/13/2016 |
| A26 | S41, S42 | *Macaca fascicularis* | Chinese | 6 | M | 5/17/2016; 5/26/2016 | 6/13/2016 |
| A27 | S43, S44 | *Macaca fascicularis* | Chinese | 6 | M | 4/5/2016; 4/22/2016 | 6/13/2016 |
| A28 | S45, S46 | *Macaca fascicularis* | Chinese | 6 | M | 5/17/2016; 5/26/2016 | 6/13/2016 |
| A29 | S47, S48 | *Macaca fascicularis* | Chinese | 6 | M | 4/5/2016; 4/21/2016 | 6/13/2016 |
| A30 | S49, S50 | *Macaca fascicularis* | Chinese | 6 | M | 5/17/2016; 5/26/2016 | 6/13/2016 |

**Table S2**: 7 core molecules identified to Level 2, according to the Metabolomics Standards Initiative. Names are putative and based on a visual comparison of their experimental spectra with library spectra (using the criteria described in the supplemental information). The forward match score shown here is that of the highest matching library hit. ^1^t̄_R_ and ^2^t̄_R_ are the mean primary and secondary retention times across all 30 test set samples for that molecule. Across samples and features, retention times used to calculate ^1^t̄_R_ and ^2^t̄_R_ have ranges of 2 s - 12 s and 0.01 s - 0.30 s, respectively. Tables S2 and S3 show the first- and second-dimension retention times of all core compounds not shown here. Those tables include compounds to which formula and/or class were assigned, as well as unknown compounds. LRI: Linear Retention Index

| **Putative name** | **Formula** | **Class** | **Forward Match Score** | **^1^t̄_R_ (s)** | **^2^t̄_R_ (s)** | **LRI** |
| --- | --- | --- | --- | --- | --- | --- |
| Toluene | C_7_H_8_ | aromatic | 928 | 936 | 0.75 | 790 |
| Decene | C_10_H_20_ | alkene | 883 | 1562 | 0.59 | 996 |
| Octanal | C_8_H_16_O | aldehyde | 942 | 1704 | 0.74 | 1044 |
| Limonene | C_10_H_16_ | terpene | 924 | 1710 | 0.66 | 1046 |
| p-Cymene | C_10_H_14_ | aromatic | 865 | 1722 | 0.72 | 1051 |
| Acetophenone | C_8_H_8_O | aromatic | 934 | 1955 | 1.19 | 1133 |
| Octanoic acid methyl ester | C_9_H_18_O_2_ | ester | 880 | 2000 | 0.71 | 1150 |

**Table S3**: Core molecules identified at Level 3 according to the Metabolomics Standard Initiative. These molecules were all assigned formula based on the criteria described in this section

| **Putative formula** | **Putative class** | **^1^t̄_R_ (s)** | **^2^t̄_R_ (s)** | **LRI** |
| --- | --- | --- | --- | --- |
| C_7_H_16_ | alkane | 612 | 0.55 | 673 |
| C_6_H_6_ | unknown | 634 | 0.73 | 683 |
| C_7_H_14_ | cycloalkane/alkene | 660 | 0.57 | 694 |
| C_7_H_16_ | alkane | 674 | 0.56 | 700 |
| C_5_H_10_O | aldehyde/ketone | 756 | 0.74 | 728 |
| C_7_H_14_ | cycloalkane | 760 | 0.57 | 730 |
| C_8_H_18_ | alkane | 768 | 0.56 | 732 |
| C_5_H_10_O | aldehyde/ketone | 774 | 0.73 | 734 |
| C_6_H_12_O | aldehyde/ketone | 907 | 0.71 | 780 |
| C_8_H_18_ | alkane | 965 | 0.57 | 800 |
| C_8_H_16_ | alkene | 1001 | 0.57 | 812 |
| C_6_H_12_O | aldehyde/ketone | 1083 | 0.75 | 838 |
| C_9_H_18_ | alkene | 1107 | 0.58 | 846 |
| C_8_H_10_ | aromatic | 1229 | 0.75 | 885 |
| C_8_H_10_ | aromatic | 1257 | 0.76 | 894 |
| C_8_H_10_ | aromatic | 1338 | 0.78 | 921 |
| C_8_H_8_ | aromatic | 1344 | 0.89 | 923 |
| C_7_H_14_O | aldehyde/ketone | 1373 | 0.76 | 933 |
| C_10_H_22_ | alkane | 1380 | 0.56 | 935 |
| C_7_H_14_O | aldehyde/ketone | 1399 | 0.75 | 941 |
| C_10_H_16_ | aliphatic hydrocarbon | 1417 | 0.60 | 947 |
| C_9_H_12_ | aromatic | 1420 | 0.73 | 948 |
| C_6_H_10_O | cycloketone | 1435 | 0.91 | 953 |
| C_9_H_12_ | aromatic | 1536 | 0.74 | 987 |
| C_9_H_12_ | aromatic | 1544 | 0.74 | 990 |
| C_8_H_16_O | aldehyde/ketone | 1552 | 0.69 | 992 |
| C_9_H_12_ | aromatic | 1560 | 0.75 | 995 |
| C_10_H_16_ | alphatic hydrocarbon | 1566 | 0.63 | 997 |
| C_6_H_8_O | cycloketone | 1574 | 1.12 | 1000 |
| C_9_H_18_O | aldehyde/ketone | 1598 | 0.66 | 1008 |
| C_9_H_14_O | aromatic | 1600 | 0.71 | 1009 |
| C_9_H_12_ | aromatic | 1599 | 0.76 | 1008 |
| C_9_H_10_ | aromatic | 1608 | 0.84 | 1011 |
| C_7_H_6_O | aromatic | 1640 | 1.28 | 1022 |
| C_9_H_12_ | aromatic | 1644 | 0.77 | 1024 |
| C_9_H_12_ | aromatic | 1737 | 0.79 | 1056 |
| C_9_H_10_ | aromatic | 1780 | 0.83 | 1070 |
| C_10_H_14_ | aromatic | 1799 | 0.72 | 1077 |
| C_10_H_14_ | aromatic | 1818 | 0.73 | 1084 |
| C_10_H_14_ | aromatic | 1853 | 0.74 | 1096 |
| C_10_H_14_ | aromatic | 1877 | 0.74 | 1104 |
| C_10_H_14_ | aromatic | 1886 | 0.75 | 1107 |
| C_10_H_14_ | aromatic | 1904 | 0.75 | 1114 |
| C_11_H_20_ | aromatic | 1965 | 0.63 | 1137 |
| C_10_H_14_ | aromatic | 2000 | 0.78 | 1150 |
| C_10_H_14_ | aromatic | 2012 | 0.78 | 1154 |
| C_9_H_12_O | aromatic | 2011 | 1.31 | 1154 |
| C_11_H_16_ | aromatic | 2098 | 0.73 | 1186 |
| C_10_H_12_ | aromatic | 2106 | 0.83 | 1189 |
| C_10_H_14_ | aromatic | 2109 | 0.80 | 1190 |
| C_12_H_26_ | alkane | 2135 | 0.59 | 1200 |
| C_10_H_8_ | aromatic | 2258 | 1.12 | 1248 |
| C_10_H_20_O_2_ | ester | 2264 | 0.71 | 1251 |
| C_12_H_18_ | aromatic | 2364 | 0.72 | 1290 |

**Table S4**: Retention times and classes of core molecules for which neither putative name nor formula could be assigned. Based on the criteria described in this section, these molecules were assigned a putative class only. 17 of them were classed as ‘unknown”

| **^1^t̄_R_ (s)** | **^2^t̄_R_ (s)** | **Class** | **LRI** |
| --- | --- | --- | --- |
| 1752 | 0.74 | alcohol | 1061 |
| 1907 | 0.83 | aldehyde/ketone | 1115 |
| 2186 | 0.81 | aldehyde/ketone | 1220 |
| 401 | 0.76 | aldehyde/ketone | 579 |
| 1676 | 0.75 | aldehyde/ketone | 1035 |
| 848 | 0.57 | alkane | 760 |
| 913 | 0.56 | alkane | 782 |
| 1209 | 0.56 | alkane | 879 |
| 1220 | 0.56 | alkane | 882 |
| 1231 | 0.56 | alkane | 886 |
| 1328 | 0.56 | alkane | 918 |
| 1445 | 0.56 | alkane | 957 |
| 1468 | 0.56 | alkane | 964 |
| 1512 | 0.56 | alkane | 979 |
| 1526 | 0.56 | alkane | 984 |
| 1569 | 0.56 | alkane | 998 |
| 1589 | 0.56 | alkane | 1005 |
| 1604 | 0.56 | alkane | 1010 |
| 1616 | 0.57 | alkane | 1014 |
| 1629 | 0.56 | alkane | 1019 |
| 1730 | 0.56 | alkane | 1053 |
| 1743 | 0.57 | alkane | 1058 |
| 1773 | 0.57 | alkane | 1068 |
| 1854 | 0.57 | alkane | 1096 |
| 2207 | 0.57 | alkane | 1228 |
| 2268 | 0.64 | alkane | 1252 |
| 2360 | 0.57 | alkane | 1289 |
| 2374 | 0.57 | alkane | 1294 |
| 2389 | 0.58 | alkane | 1300 |
| 2531 | 0.58 | alkane | 1359 |
| 2562 | 0.58 | alkane | 1372 |
| 2576 | 0.58 | alkane | 1378 |
| 2604 | 0.60 | alkane | 1390 |
| 2628 | 0.59 | alkane | 1400 |
| 2713 | 0.58 | alkane | 1440 |
| 2843 | 0.57 | alkane | 1500 |
| 2848 | 0.60 | alkane | 1502 |
| 3063 | 0.60 | alkane | 1599 |
| 3162 | 0.59 | alkane | 1648 |
| 3265 | 0.60 | alkane | 1700 |
| 1155 | 0.57 | alkene | 861 |
| 2011 | 1.36 | aromatic | 1154 |
| 2512 | 0.70 | carboxylic acid methyl ester | 1351 |
| 2965 | 0.70 | carboxylic acid methyl ester | 1555 |
| 3369 | 0.70 | carboxylic acid methyl ester | 1755 |
| 1826 | 0.67 | chloroalkane | 1086 |
| 2124 | 0.60 | cycloalkane/alkene | 1196 |
| 648 | 0.55 | unknown | 689 |
| 826 | 0.57 | unknown | 752 |
| 1012 | 0.57 | unknown | 815 |
| 1515 | 0.74 | unknown | 980 |
| 1663 | 0.81 | unknown | 1030 |
| 1669 | 0.70 | unknown | 1032 |
| 1739 | 0.76 | unknown | 1056 |
| 1910 | 1.13 | unknown | 1116 |
| 1930 | 0.79 | unknown | 1124 |
| 1956 | 1.57 | unknown | 1133 |
| 1992 | 1.10 | unknown | 1147 |
| 2070 | 0.68 | unknown | 1176 |
| 2400 | 0.53 | unknown | 1305 |
| 2652 | 0.58 | unknown | 1411 |
| 3007 | 0.59 | unknown | 1574 |
| 3169 | 0.69 | unknown | 1652 |
| 3314 | 0.71 | unknown | 1725 |

**Table S5**: The 17 compounds for which a match in putative molecular formulae or putative name was found between this study and reference 30 (Bishop et al., 2019) were found.

| **Putative name/molecular formula** |
| --- |
| Octanal |
| o-cymene |
| C_10_H_12_ |
| C_10_H_14_ |
| C_10_H_16_ |
| C_10_H_22_ |
| C_11_H_16_ |
| C_12_H_26_ |
| C_5_H_10_O |
| C_6_H_10_O |
| C_6_H_12_O |
| C_7_H_14_O |
| C_8_H_10_ |
| C_8_H_16_ |
| C_8_H_16_O |
| C_9_H_18_ |
| C_9_H_18_O |

**Table S6**: The distribution of core features found in the training set but absent in the validation set. A **black cell** represents an instance of a feature missing in the corresponding validation sample

| ID | Core Features Not Found  (mean retention times in seconds) | | | | | | | | | | | |
| --- | --- | --- | --- | --- | --- | --- | --- | --- | --- | --- | --- | --- |
|  | C_5_H_10_O  (756,0.74) | Alkane  (1220,0.56) | Alkane  (1231,0.56) | Alkane  (1569,0.56) | C_9_H_18_O  (1598,0.66) | Unknown  (1669,0.70) | C_10_H_14_  (1818,0.73) | Unknown  (1910,1.13) | Unknown  (1930,0.79) | Unknown  (1956,1.57) | Unknown  (1992,1.10) | Alkane  (2843,0.57) |
| S4 |  |  |  |  |  |  |  |  |  |  |  |  |
| S14 |  |  |  |  |  |  |  |  |  |  |  |  |
| S24 |  |  |  |  |  |  |  |  |  |  |  |  |
| S26 |  |  |  |  |  |  |  |  |  |  |  |  |
| S40 |  |  |  |  |  |  |  |  |  |  |  |  |
| S42 |  |  |  |  |  |  |  |  |  |  |  |  |

**Figure S1**: (A) A violin plot illustrating the average PQN normalized peak area of each analyte by volatilome class. A Wilcox rank sum test was used to test if the average PQN normalized peak area were different across volatilome classes. (B) The density distribution of the variance in normalized peak area of analytes in the core volatilome. The threshold cut-off of a variance <0.05 is indicated for the critical core analytes.

**(B)**

**(A)**


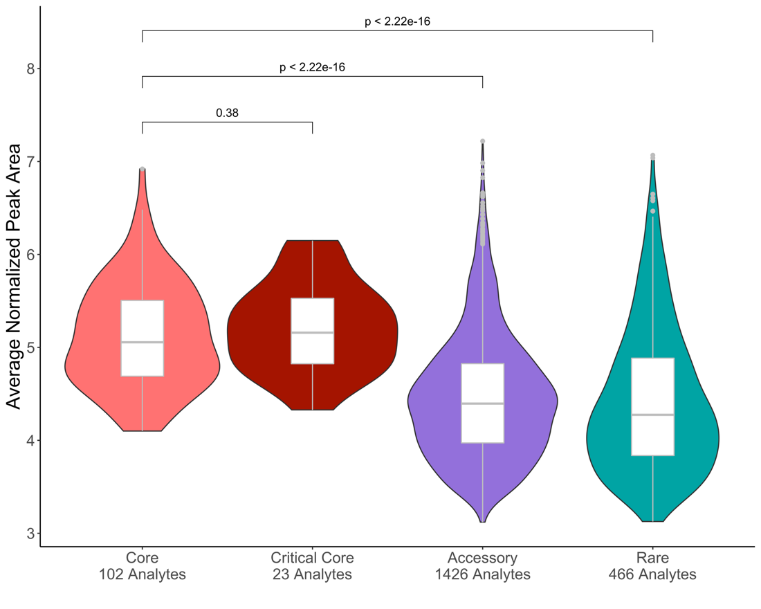

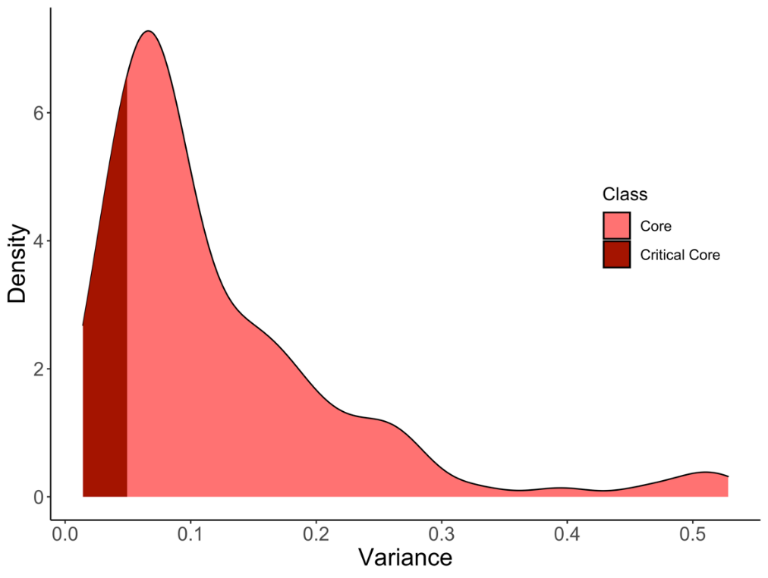

Supplement: Supplementary file 1 — Supplementary Information. [file 41598_2024_64910_MOESM1_ESM.docx]
